# Supplementary figures and images for: A feature of maternal sleep apnea during gestation causes autism-relevant neuronal and behavioral phenotypes in offspring
Source: PLoS Biol. 2022 Feb 3;20(2):e3001502. doi: 10.1371/journal.pbio.3001502 (PMC8812875; doi:10.1371/journal.pbio.3001502)

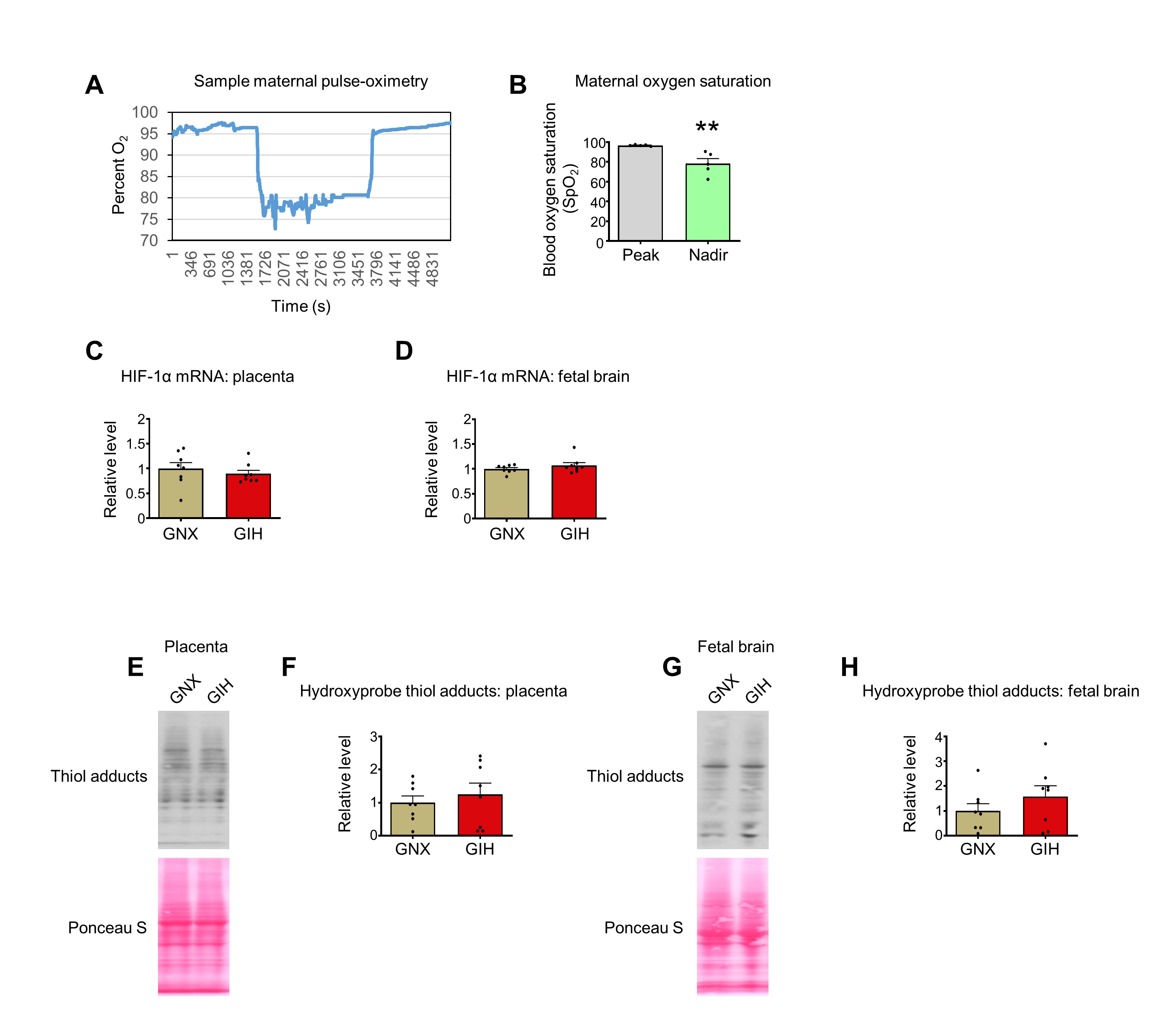

Supplement: S1 Fig — (A) Representative pulse oximetry trace in a pregnant rat dam in response to the cyclic oxygen fluctuations of GIH. (B) Graph shows the average nadir and average peak in oxyhemoglobin saturation for 5 pregnant rat dams subjected to the cyclic oxygen fluctuations of GIH. Oxyhemoglobin saturation levels reach a mean nadir of 78% at the height of hypoxia, with a mean 97% saturation level achieved between nadirs (p = 0.0068). n = 5 pregnant rat dams. (C) No differences in HIF-1α mRNA levels were detected between GNX and GIH offspring placentas [t (df,14) = 0.7529, p = 0.4640]. n = 8 GNX and 8 GIH placentas. (D) No differences in HIF-1α mRNA levels were detected between GNX and GIH offspring fetal brains [t (df,14) = 1.138, p = 0.2744]. n = 8 GNX and 8 GIH fetal brains. (E) Blots show levels of protein thiol adducts and total protein (Ponceau S) in GNX and GIH placentas. (F) Quantification revealed no differences in protein thiol adduct levels in the placentas of GNX versus GIH offspring [t (df,14) = 0.6192, p = 0.5457]. n = 8 GNX and 8 GIH placentas. (G) Blots show levels of protein thiol adducts and total protein (Ponceau S) in GNX and GIH fetal whole brain homogenates. (H) Quantification revealed no differences in protein thiol adduct levels in fetal whole brain homogenates of GNX versus GIH offspring [t (df,14) = 1.114, p = 0.2839]. n = 8 GNX and 8 GIH fetal brains. All bar graphs are the mean + SEM. The data underlying this figure can be found in S1 Raw Data. GIH, gestational intermittent hypoxia; GNX, gestational normoxia; HIF-1α, hypoxia-inducible factor 1α. (TIF) [file pbio.3001502.s001.TIF]

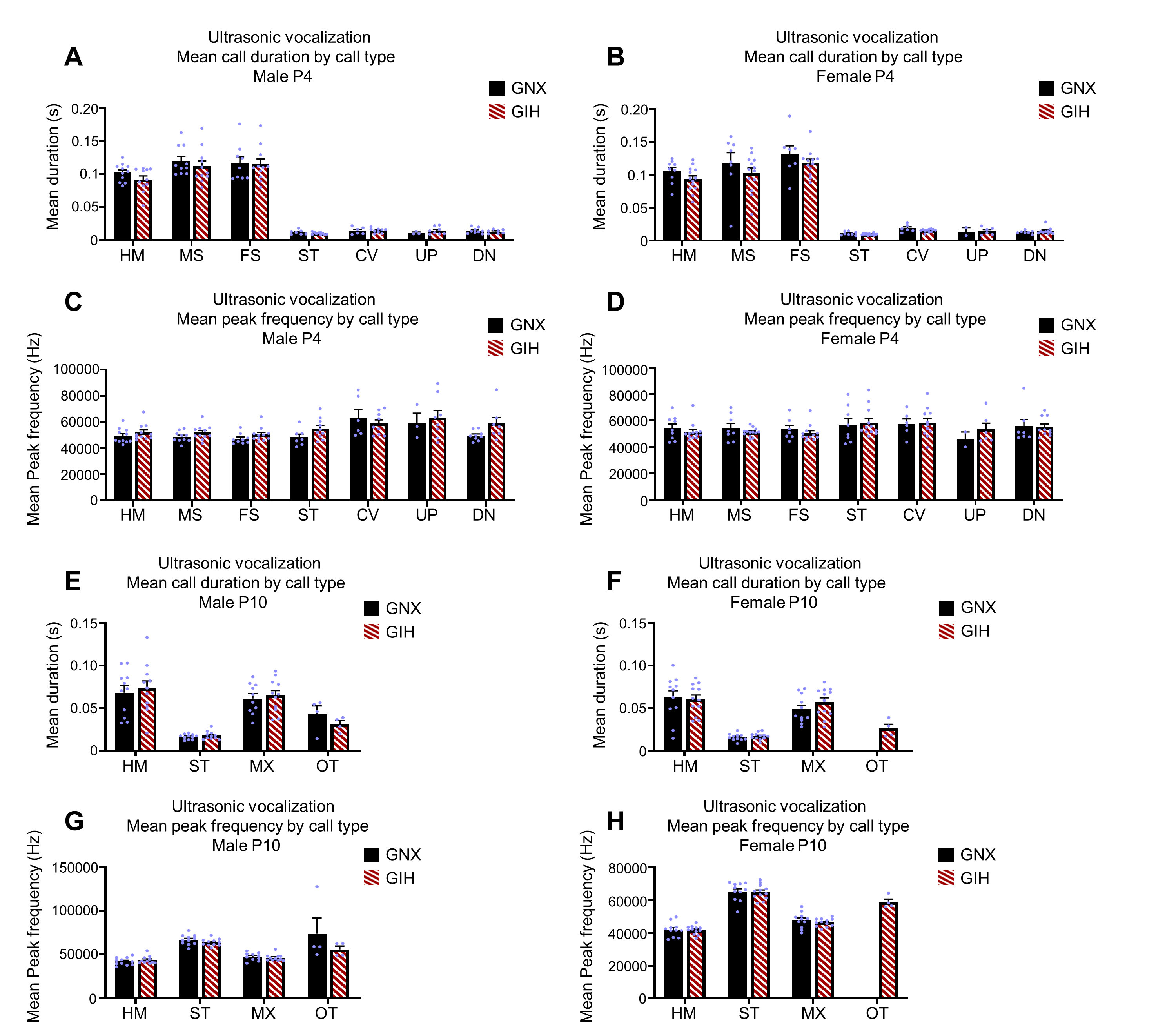

Supplement: S2 Fig — (A) No differences in mean duration of individual USV call types were detected between male P4 GIH and GNX offspring. [False discovery correction post hoc (df,108): HM q = 0.9789; MS, FS, ST, CV, UP, and DN q = 1.0] n = 11 GNX and 11 GIH rats. (B) No differences in mean duration of individual USV call types were detected between female P4 GIH and GNX offspring. [False discovery correction post hoc (df,110): HM, MS, FS q = 0.3886; ST, CV, UP, and DN q = 1.0] n = 9 GNX and 13 GIH rats. (C) No differences in mean peak frequency of individual USV call types were detected between male P4 GIH and GNX offspring. [False discovery correction post hoc (df,108): HM, MS, FS q = 0.5148; ST q = 0.3959; CV q = 0.5148; UP q = 0.5148; DN q = 0.2196] n = 11 GNX and 11 GIH rats. (D) No differences in mean peak frequency of individual USV call types were detected between female P4 GIH and GNX offspring. [False discovery correction post hoc (df,110): HM, MS, FS, ST, CV, UP, and DN q = 0.9430] n = 9 GNX and 13 GIH rats. (E) No differences in mean duration of individual USV call types were detected between male P10 GIH and GNX offspring. [False discovery correction post hoc (df,65): HM, ST, MX, OT q = 0.8976] n = 11 GNX and 11 GIH rats. (F) No differences in mean duration of individual USV call types were detected between female P10 GIH and GNX offspring. [False discovery correction post hoc (df,63): HM q = 0.8915; ST q = 0.8915; MX q = 0.6432; OT q = n/a] n = 11 GNX and 12 GIH rats. (G) No differences in mean peak frequency of individual USV call types were detected between male P10 GIH and GNX offspring. [False discovery correction post hoc (df,65): HM, ST, MX, OT q = 0.6009] n = 11 GNX and 11 GIH rats. (H) No differences in mean peak frequency of individual USV call types were detected between female P10 GIH and GNX offspring. [False discovery correction post hoc (df,63): HM, ST, MX q = 0.9101; OT q = n/a] n = 11 GNX and 12 GIH rats. All bar graphs are the mean + SEM. The data underlyi [file pbio.3001502.s002.TIF]

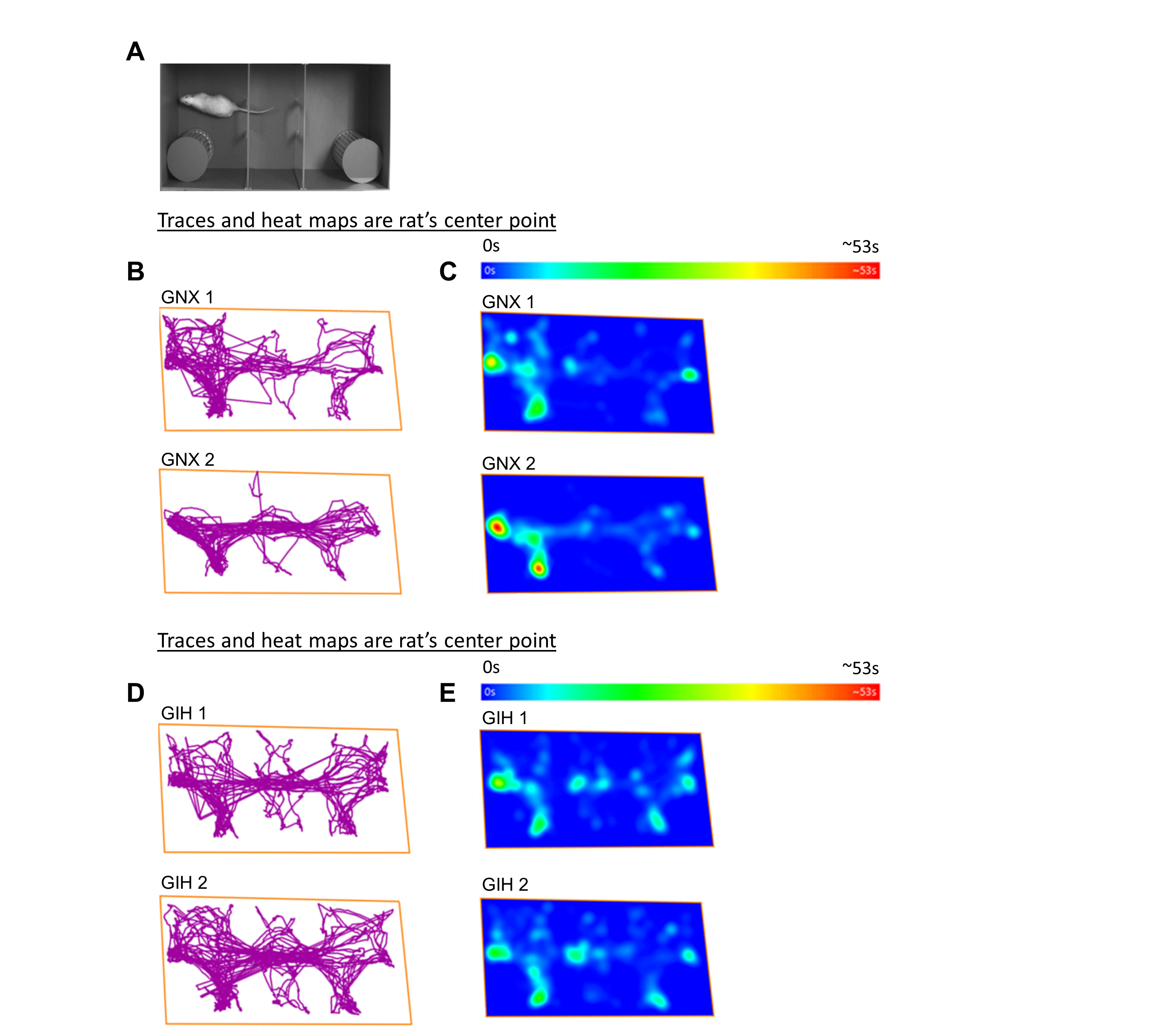

Supplement: S3 Fig — (A) Picture of the 3-chambered arena used for social approach testing. (B and C) Sample traces and heat maps from 2 GNX male offspring. The traces and heat maps are from the rat’s center point. (D and E) Sample traces and heat maps from 2 GIH male offspring. The traces and heat maps are from the rat’s center point. GIH, gestational intermittent hypoxia; GNX, gestational normoxia. (TIF) [file pbio.3001502.s003.TIF]

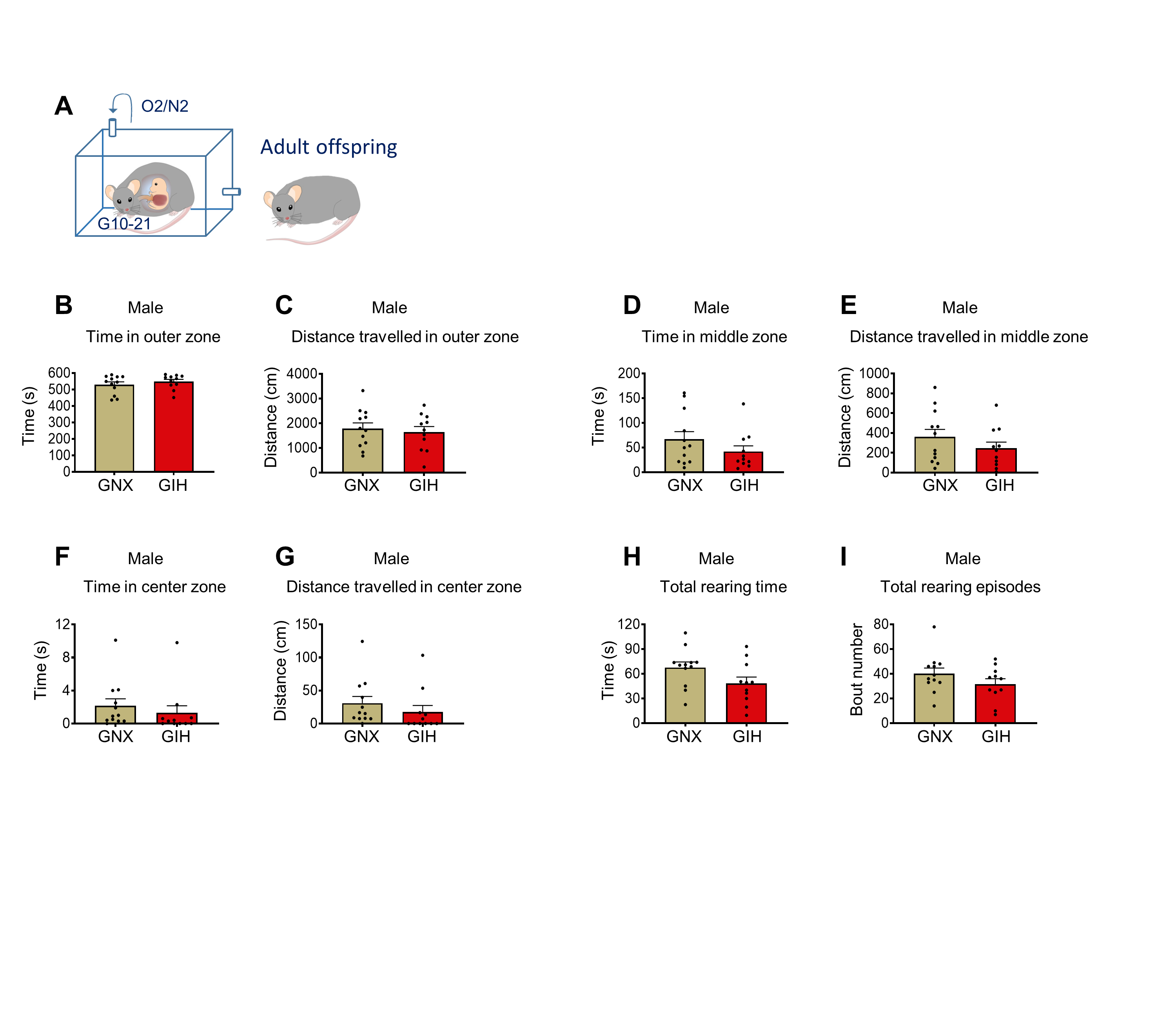

Supplement: S4 Fig — (A) Schematic depicting offspring age pertaining to all data in S4 Fig. (B and C) No differences in adult GIH male offspring were detected for time [t (df,21) = 0.8508, p = 0.4047] or distance traveled [t (df,21) = 0.4672 = 0.6451] in the outer zone of the open field. n = 12 GNX and 11 GIH rats. (D and E) No differences in adult GIH male offspring were detected for time [t (df,21) = 1.261, p = 0.2211] or distance traveled (t (df,21) = 1.142, p = 0.2663] in the middle zone of the open field. n = 12 GNX and 11 GIH rats. (F and G) No differences in adult GIH male offspring were detected for time [t (df,21) = 0.7341, p = 0.4697] or distance traveled [t (df,21) = 0.9210, p = 0.3675] in the center zone of the open field. n = 12 GNX and 11 GIH rats. (H and I) No differences in adult GIH male offspring were detected for total rearing time [t (df,32) = 1.887, p = 0.0731] or total rearing episodes [t (df,21) = 1.345, p = 0.1928] in the open field. n = 12 GNX and 11 GIH rats. All bar graphs are the mean + SEM. The data underlying this figure can be found in S1 Raw Data. GIH, gestational intermittent hypoxia; GNX, gestational normoxia. (TIF) [file pbio.3001502.s004.TIF]

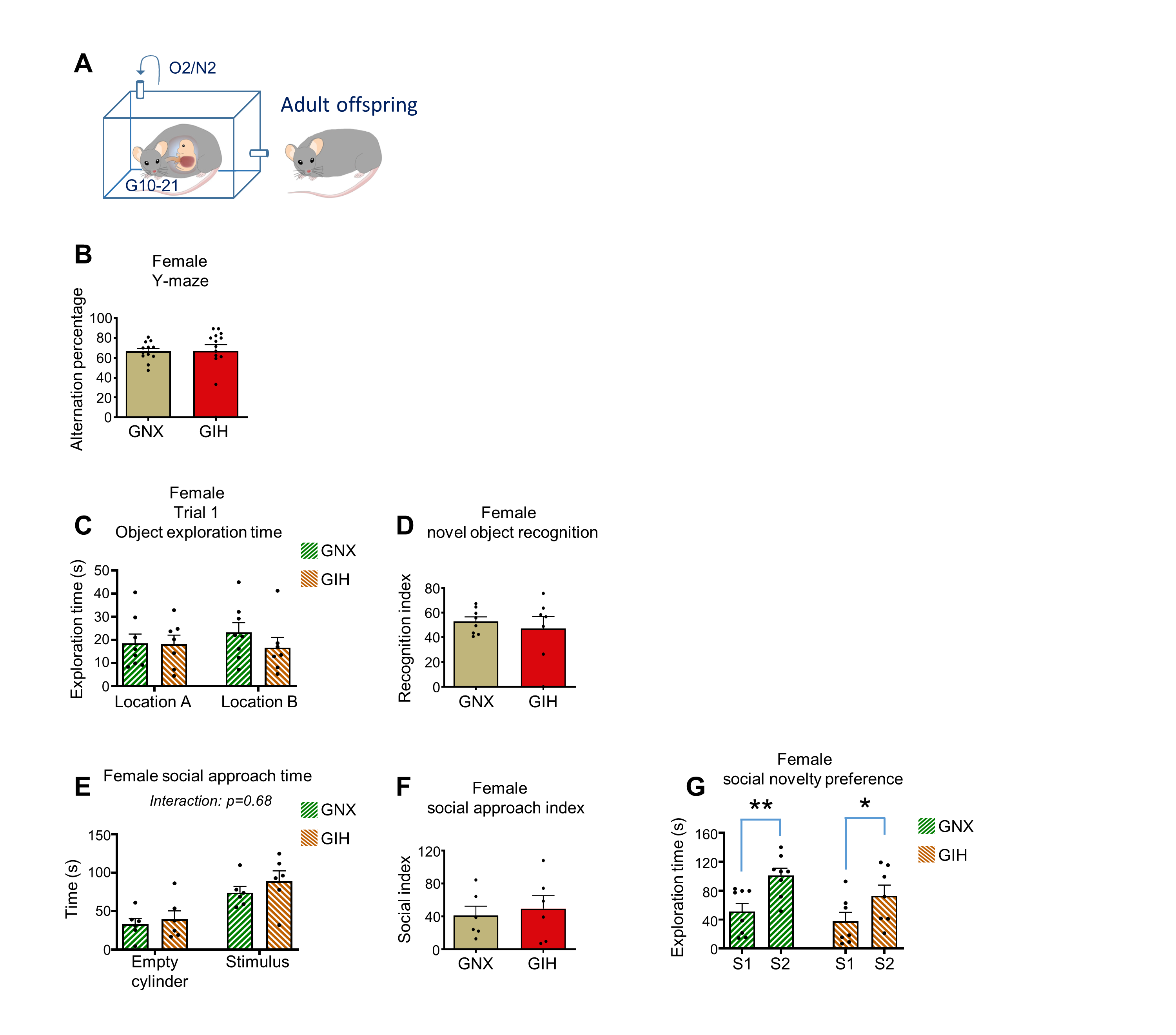

Supplement: S5 Fig — (A) Schematic depicting offspring age pertaining to all data in S5 Fig. (B) Analysis of mean Y-maze spontaneous alternation percentage in adult female GNX and GIH offspring revealed no differences [t (df,24) = 0.0444, p = 0.9649]. n = 12 GNX and 14 GIH rats. (C) Object exploration time during trial 1 of the object recognition test revealed no differences in time spent investigating either object location in GNX and GIH female offspring [Bonferroni post hoc (df,26) GNX p = 0.8395, GIH p = 1.0]. n = 8 GNX and 7 GIH rats. (D) Assessment of mean novel object recognition index revealed no differences between adult female GIH versus GNX [t (df,13) = 0.5896, p = 0.5656]. n = 8 GNX and 7 GIH rats. (E) Time spent investigating each cylinder in the social approach task for female adults. GIH and GNX adult female offspring spent similar time investigating the stimulus cylinder [Bonferroni post hoc (df,20) p = 0.6124], with no interaction of maternal GNX/GIH status by end chamber cylinder detected [F(1,10) = 0.1860, p = 0.6754]. (F) Social approach index assessment revealed no differences between adult female GIH versus GNX [t (df,10) = 0.4313, p = 0.6754]. n = 6 GNX and 6 GIH rats. (G) Social recognition analysis indicates that both adult female GNX and female GIH offspring spend more time investigating the novel (S2) versus familiar (S1) rat [GNX Bonferroni post hoc (df,13) p = 0.0010; GIH Bonferroni post hoc (df,13) p = 0.0183]. n = 8 GNX and 7 GIH rats. All bar graphs are the mean + SEM. The data underlying this figure can be found in S1 Raw Data. GIH, gestational intermittent hypoxia; GNX, gestational normoxia. (TIF) [file pbio.3001502.s005.TIF]

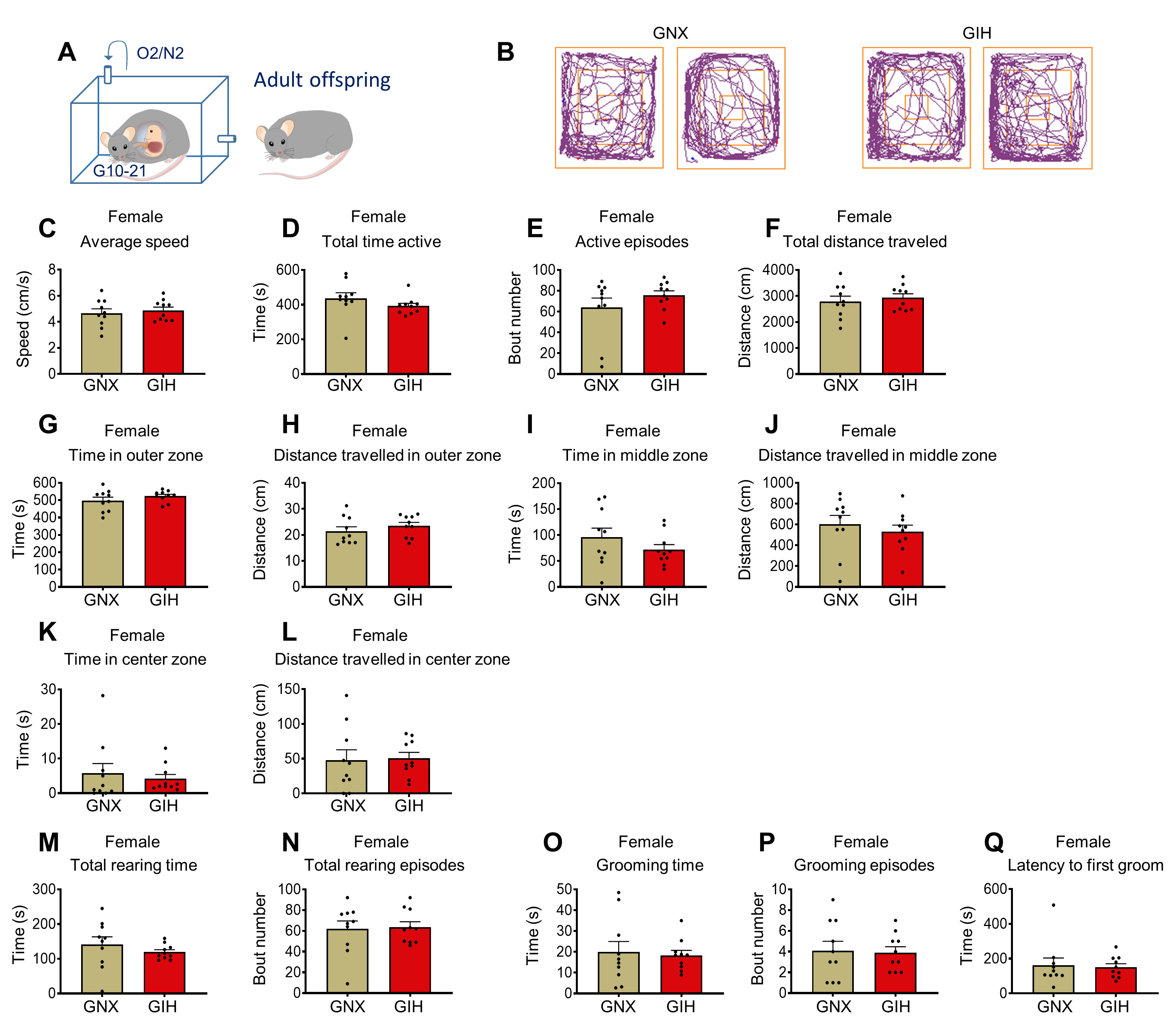

Supplement: S6 Fig — (A) Schematic depicting offspring age pertaining to all data in S6 Fig. (B) Sample locomotor traces in an open field across 10 minutes for adult female GNX and GIH offspring. Outer, middle, and inner zones of the open field are shown in orange. (C–F) No differences in mean speed in the open field (C), total time active (D), number of active episodes (E), or total distance traveled (F) were detected between adult female GNX and GIH offspring [(df,18), avg speed t = 0.5261, p = 0.6053; time active t = 1.270, p = 0.2201; active episodes t = 1.182, p = 0.2525; distance traveled t = 0.5532, p = 0.5869]. n = 10 GNX and 10 GIH rats. (G and H) No differences in adult GIH female offspring were detected for time [t (df,18) = 1.150, p = 0.2652] or distance traveled (t (df,18) = 0.9712, p = 0.3443) in the outer zone of the open field. n = 10 GNX and 10 GIH rats. (I and J) No differences in adult GIH female offspring were detected for time (t (df,18) = 1.179, p = 0.2538) or distance traveled [t(18) = 0.6521, p = 0.5226] in the middle zone of the open field. n = 10 GNX and 10 GIH rats. (K and L) No differences in adult GIH female offspring were detected for time [t (df,18) = 0.5125, p = 0.6147] or distance traveled [t (df,18) = 0.1628, p = 0.8725] in the center zone of the open field. n = 10 GNX and 10 GIH rats. (M and N) No differences in adult GIH female offspring were detected for total rearing time [t (df,18) = 0.9740, p = 0.3430] or total rearing episodes [t (df,18) = 0.1747, p = 0.8632] in the open field. n = 10 GNX and 10 GIH rats. (O–Q) No differences in adult GIH female offspring were detected for total grooming time (t (df,18) = 0.2892, p = 0.7758), number of grooming episodes [t (df,18) = 0.1881, p = 0.8532], or latency to the first grooming episode [t (df,18) = 0.2531, p = 0.8031]. n = 10 GNX and 10 GIH rats. All bar graphs are the mean + SEM. The data underlying this figure can be found in S1 Raw Data. GIH, gestational intermittent hypoxia; GNX, gestational normoxia. [file pbio.3001502.s006.TIF]

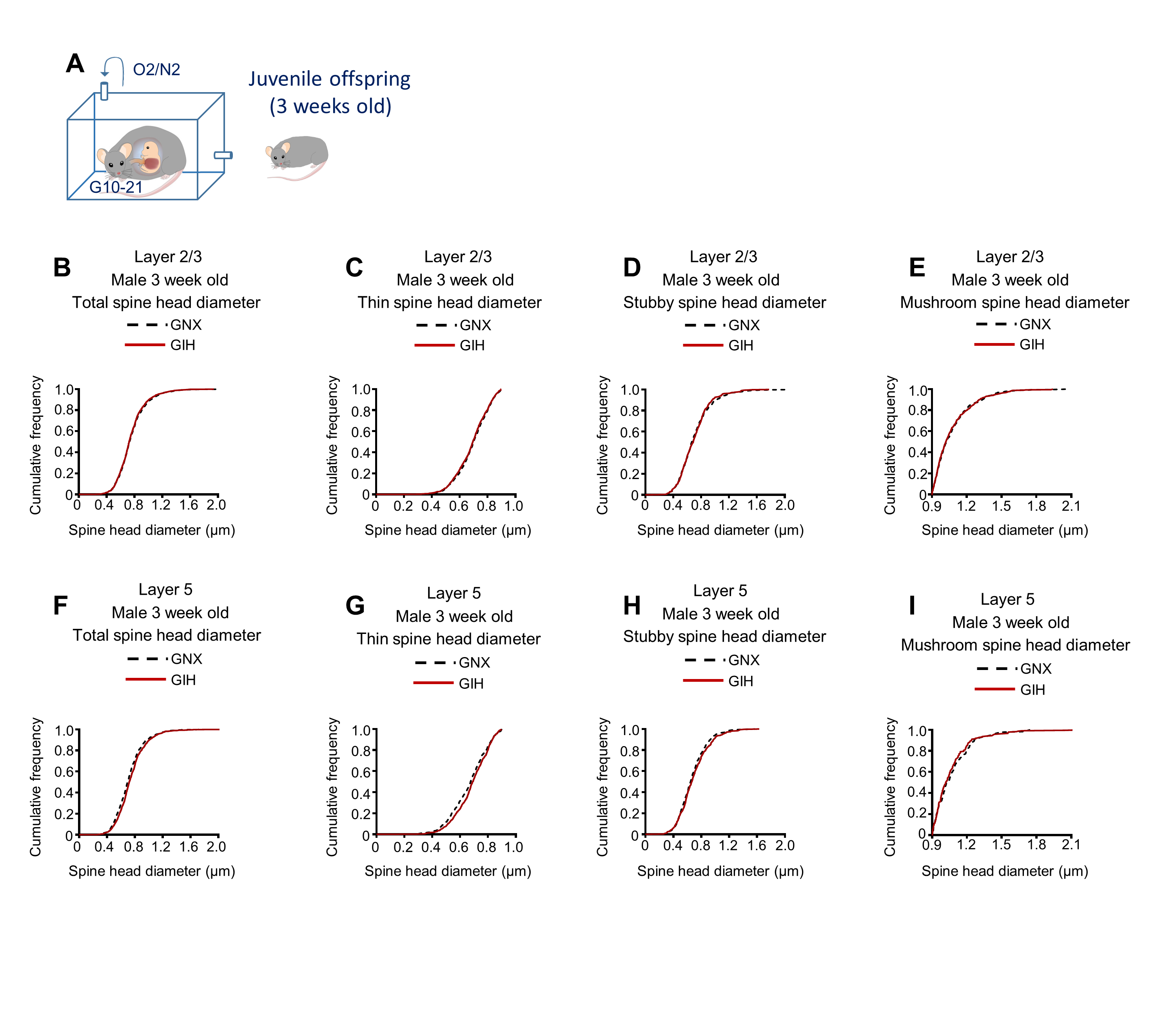

Supplement: S7 Fig — (A) Schematic depicting offspring age pertaining to all data in S7 Fig. (B–E) No significant differences in layer 2/3 total or layer 2/3 spine subtype cumulative head diameter curves were identified between 3-week-old GIH and GNX offspring (total spine median survival differential 0.86%, thin differential 0.86%, stubby differential 3.2%, and mushroom differential 0.56%). n = 2,778 GNX and 2,636 GIH total spines; 1,712 GNX and 1,740 GIH thin spines; 603 GNX and 470 GIH stubby spines; 463 GNX and 426 GIH mushroom spines. (F–I) No significant differences in layer 5 total or layer 5 spine subtype cumulative head diameter curves were identified between 3-week-old GIH and GNX offspring (total spine median survival differential 3.3%, thin differential 2.3%, stubby differential 2.8%, and mushroom differential 0.36%). n = 1,377 GNX and 1,484 GIH total spines; 915 GNX and 935 GIH thin spines; 287 GNX and 313 GIH stubby spines; 175 GNX and 236 GIH mushroom spines. The data underlying this figure can be found in S1 Raw Data. GIH, gestational intermittent hypoxia; GNX, gestational normoxia; mPFC, medial prefrontal cortex. (TIF) [file pbio.3001502.s007.TIF]

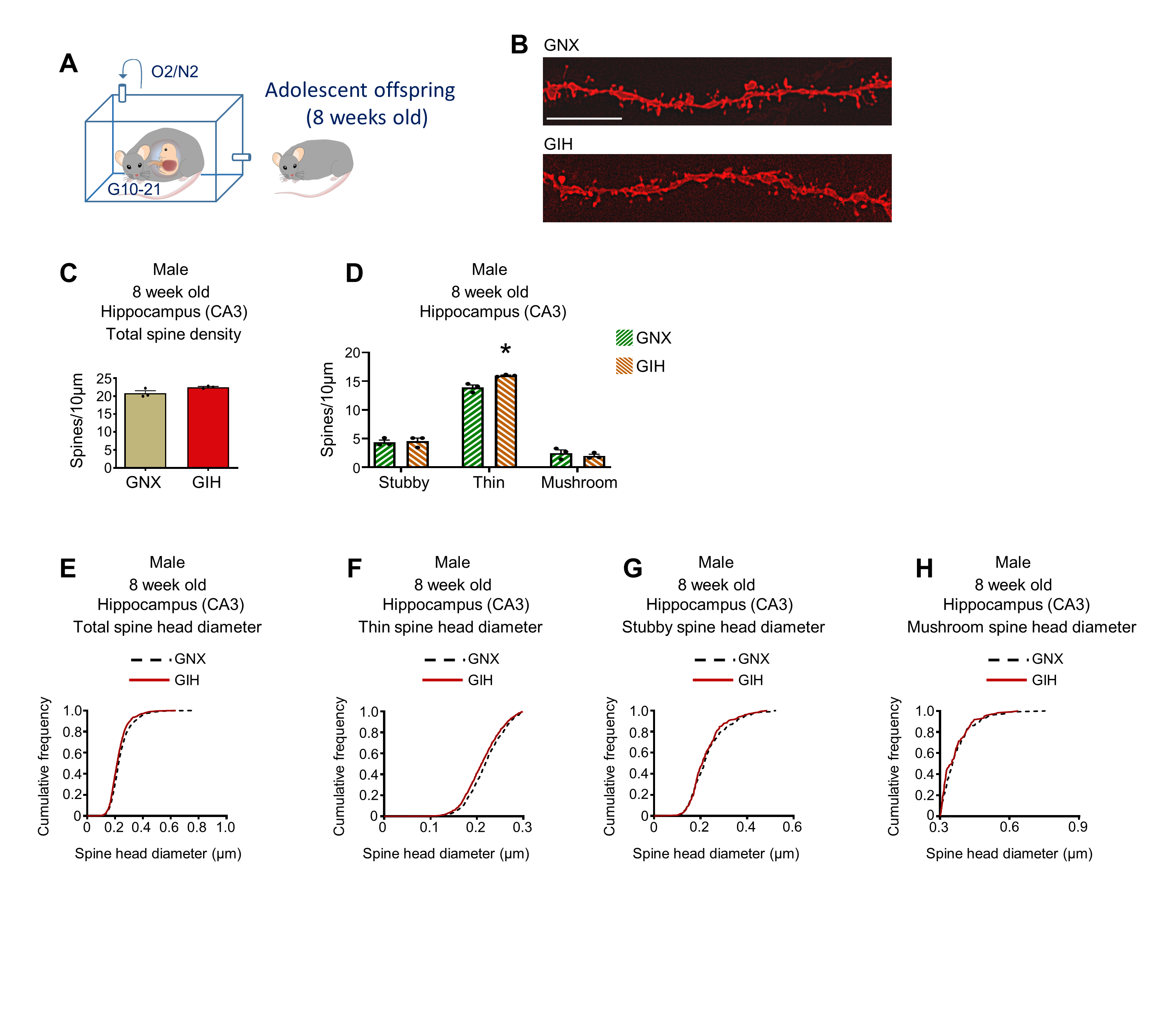

Supplement: S8 Fig — (A) Schematic depicting offspring age pertaining to all data in S8 Fig. (B) Representative hippocampal CA3 field pyramidal neuron dendrite segments from 8-week-old male GNX and GIH offspring. Scale bar = 5 μm. (C) No significant difference in total CA3 spine density was detected between GIH and GNX offspring [t (df,4) = 2.301, p = 0.0829]. n = 3 GNX and 3 GIH rats. (D) CA3 spine subtype analysis revealed a significant increase in the density of thin spines [Bonferroni post hoc (df,12), p = 0.0130] in GIH offspring relative to GNX offspring, with no changes in the density of stubby [Bonferroni post hoc (df,12), p = 1.0] or mushroom spines [Bonferroni post hoc (df,12), p = 1.0]. n = 3 GNX and 3 GIH rats. (E–H) Spine head diameter curves for all spines (E) and for thin (F), stubby (G), and mushroom spines (H). No differences in head diameter curves between GIH and GNX offspring were detected for all spines combined or for any spine subtypes (total spine median survival differential 4.62%, thin differential 3.8%, stubby differential 3.52%, and mushroom differential 0.63%). n = 1,528 GNX and 1,439 GIH total spines; 1,010 GNX and 1,032 GIH thin spines; 319 GNX and 285 GIH stubby spines; 199 GNX and 122 GIH mushroom spines. All bar graphs are the mean + SEM. The data underlying this figure can be found in S1 Raw Data. GIH, gestational intermittent hypoxia; GNX, gestational normoxia. (TIF) [file pbio.3001502.s008.TIF]
