# Supplementary material for: A feature of maternal sleep apnea during gestation causes autism-relevant neuronal and behavioral phenotypes in offspring
Source: PLoS Biol. 2022 Feb 3;20(2):e3001502. doi: 10.1371/journal.pbio.3001502 (PMC8812875; doi:10.1371/journal.pbio.3001502)

Uncropped Western Blots pertaining to main figure 8

- Prior to probing for any proteins, the entire blot was cut horizontally at about 80kda and 32kda to give 3 blots (high, mid, and low molecular weight containing blots).
- kda listed in parenthesis is the known molecular weight for each indicated protein – we assured that the same weight band appeared and was cropped for corresponding phopsho and total proteins.
- Blue arrow is the cropped band shown in main figure 8 and red rectangle the cropped lanes shown.

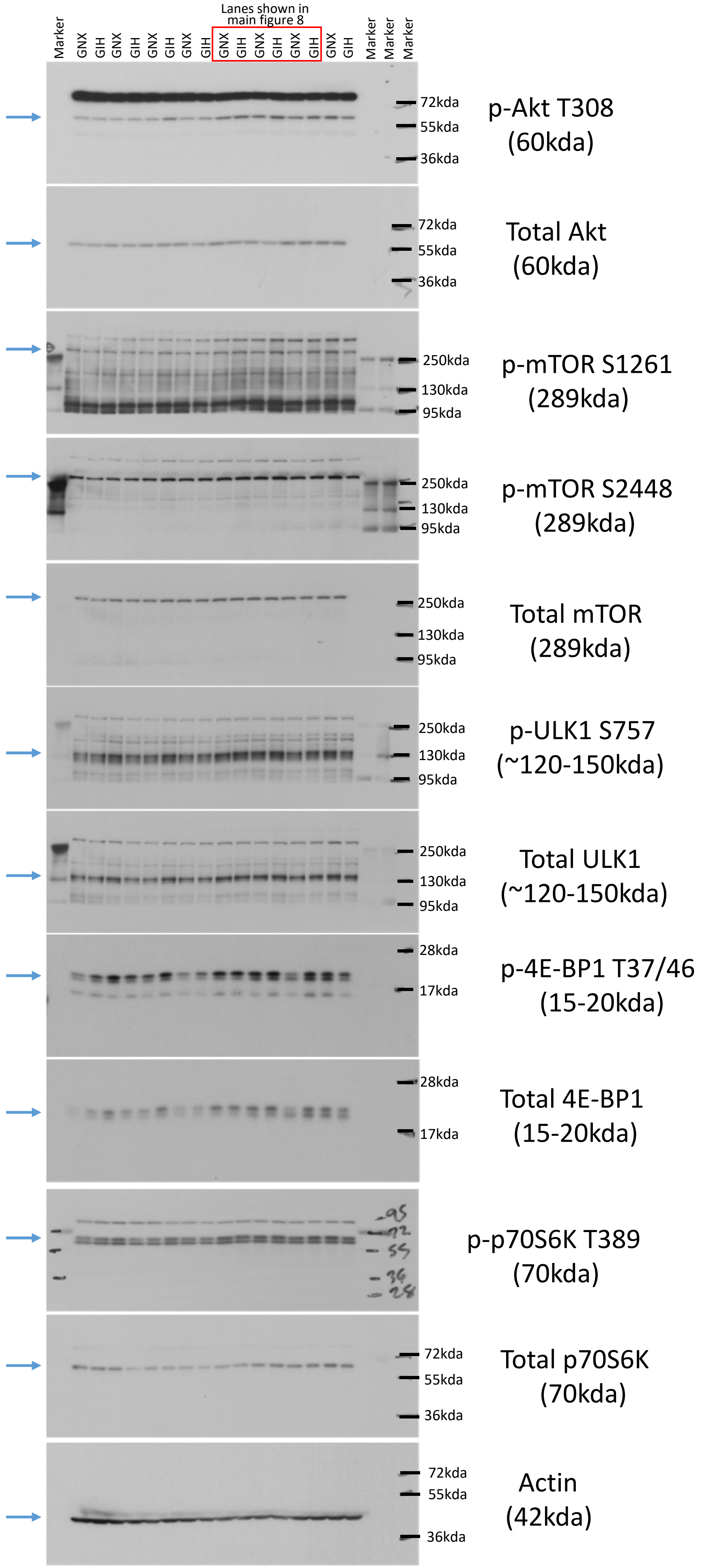

Supplement: S1 Raw Images — (PDF) [file pbio.3001502.s009.pdf]
