# Supplementary material for: A feature of maternal sleep apnea during gestation causes autism-relevant neuronal and behavioral phenotypes in offspring
Source: PLoS Biol. 2022 Feb 3;20(2):e3001502. doi: 10.1371/journal.pbio.3001502 (PMC8812875; doi:10.1371/journal.pbio.3001502)
Supplement: Attachment — Submitted filename: Response to Reviewers.pdf [file pbio.3001502.s011.pdf]

REVIEWS:

Reviewer #1: This interesting manuscript addresses the intriguing hypothesis that maternal sleep apnea during pregnancy produces a risk for autism. Literature is cited for a steep rise in the incidence of sleep apnea during the third trimester of pregnancy, particularly in obese mothers, and for associations of maternal sleep apnea with psychiatric and neurodevelopmental disorders including autism in their children.

The authors developed a rat model in which pregnant rats were exposed to intermittent hypoxia, involving cyclic bouts of 2 minutes of 10.5% oxygen, throughout 8 hours of their sleep cycle, during the second half of gestation. Several biological outcome measures were investigated: litter size and survival, gene expression of the hypoxia-inducible factor 1α (HIF-1α) in brain and placenta, dendritic spine density and morphology in medial prefrontal cortex, and AMPA-mediated spontaneous excitatory postsynaptic currents in pyramidal neurons. Behavioral consequences were tested on an appropriate sequence of rat behavioral assays relevant to the symptoms of autism spectrum disorder: ultrasonic distress vocalizations emitted by separated pups, Y-maze spontaneous alternation, novel object recognition (24 hour interval), 3-chambered sociability and social recognition. Results reveal that intermittent prenatal hypoxia led to male and female pups emitting more separation-induced vocalizations at postnatal days 4 and 10. Juveniles and adults displayed impaired cognition and social deficits, primarily in males. Further, dendritic spines densities were higher in the prenatal hypoxia groups, with differences in spine shapes.

These studies represent an excellent set of multidisciplinary experiments across the domains of general health, pup and adult behaviors, electrophysiology, and synaptic morphology. Appropriate statistical analyses were conducted throughout. Ns of 9-13 rats per sex and per treatment group were employed. While N=9 is somewhat low for behavioral assays, results appear to be robust, and replicated at two ages. Results are well presented in clear graphs, with raw data shown in Supplementary. The Discussion section is well written in terms of summarizing the data and appropriately interpreting the results. The authors are to be congratulated on their excellent studies.

We appreciate the enthusiasm for our work.

Minor changes are required to improve the manuscript:

1. Please add definitions of GIH and GNX to legends of main figure. Readers will appreciate the reminders, since the abbreviations are not self-explanatory.

We have now added the definitions of these abbreviations in the description of panel A for all of the main figures.

2. Please add more specific descriptions of the glass and plastic novel objects, including dimensions.

We have now added the description of the objects used for novel object recognition to the Methods section under the 'novel object recognition' subheading.

3. Social approach data should be shown as time spent exploring the novel object (wire cage) and time spent exploring the novel mouse. Please add this graph and its associated statistics to the main and supplementary figures. This graphical presentation and statistical comparison are correctly shown for social recognition in Figure 2 Panel J. The same should be done for the sociability phase of the assay.

In our first submission, we evaluated social approach behavior by calculating a social approach index defined as the amount of time investigating the cylinder containing a novel rat minus time spent investigating the empty cylinder. To further expand this analysis, as requested we have now included graphs showing the social approach data as time spent exploring the mesh cylinder in each end

chamber. These new graphs are contained in Main Figures 2H, 2I, 3E, and Supplementary Figure 5E. The statistics for these new graphs are contained in the text as well as in the applicable figure legends. Overall, this new analysis confirms the findings from our initial social index analysis, as GIH juvenile and adolescent male offspring show a reduction in time spent investigating the cylinder containing the stimulus rat relative to their male GNX counterparts, with no differences detected between female GIH and GNX rats at any stage of postnatal development.

4. Note that the term "preference for social novelty" is more correct than "social recognition" for the last phase of the 3-chambered assay. Bone fide social recognition is an assay involving 4 presentations of the same partner, followed by 1 presentation of a new partner. It would be best to change to "preference for social novelty" throughout.

We agree that our prior used of the term social recognition is misleading. We have now used preference for social novelty when describing these data both in the main text and in the applicable figure legends.

5. Methods for the 3-chambered test describe 15 cm diameter wire cages in an arena that measures 80 cm total length x 40 cm width. The container cage therefore is taking up approximately half the length and one third the width of its side chamber. This test usually requires more room for the subject rat to explore the entire arena, and choose to explore the novel object and the novel mouse. Possibly the available equipment was built for mice. For future experiments, the authors are encouraged to have a larger 3-chambered apparatus built for rats.

The 3-chambered social arena and mesh cylinders were purchased from Maze Engineers (Skokie, IL), and the size of the arena and cylinders was designed by this company for testing rats. We have now included a picture of the social arena and cylinder used, and representative traces and heat maps of GNX and GIH male offspring during the social approach task -- now shown in Supplementary Figure 3. In performing additional experiments for the revision process, we found that our deficit in social approach behavior in GIH male offspring compared to GNX male offspring is fully reproducible using this equipment (we tested additional rats when investigating a biochemical mechanism for our findings, which reproduced our prior findings of a deficit in GIH males). Because of this, we do not feel that the arena used affected our ability to assess social approach behavior. However, we appreciate that it would be ideal to use a larger arena for future studies to enable the rats to have more freedom of exploration, and will make this adjustment going forward.

6. Page 27 states that the experimenter was blinded to the two treatment conditions. Please add a detailed description of exactly how the experimenter was kept uninformed of treatment condition when scoring videos.

We have now elaborated on the blinding procedure in the 'behavior testing' subsection under the 'Method Details' heading. As now stated, for blinding, rats were ear tagged with arbitrary identification numbers by an investigator in a different lab than that performing the behavioral testing and analysis. This individual, who was not involved in the study, assigned rats to another experimenter for behavioral testing to assure roughly equal numbers of rats per condition would be assessed for each behavioral task. The experimental group of individual identification numbers was not available to the investigator who performed the behavioral tasks and those who performed the subsequent analysis until after behavioral scoring was completed.

7. References to the behavioral testing methods cite review articles rather than methods papers. Please add references to key research papers that describe the actual methods employed in the present studies, for each behavioral assay.

For each behavioral test, have now added citations to primary research papers that guided the methods used in our experiments. These citations are included in the main text when first introducing each behavioral task.

8. Could the authors speculate on whether the consequences of prenatal intermittent hypoxia were more likely the direct result of (a) lower oxygen during late gestation, versus (b) the presumably elevated activity of the GIH dams when awoken by apnea episodes, (c) the presumed lower amount of sleep in the awakened GIH dams, and/or (d) impairments in subsequent maternal behaviors of the GIH dams?

We have now expanded on a paragraph in the Discussion section (on pages 27-28) in which we address these issues. In summary, as we do not find evidence for hypoxia in the placenta or fetus in the GIH condition, we do not think this is a contributor of the male phenotypes – data shown in Supplementary Figure 1. In terms of activity and sleep disruption of the mothers as a possible contributor, we also do not think these are major factors as a previous study using gestational intermittent hypoxia characterized the sleep behavior of the mothers and found a disruption in the sleep cycle during the first day of exposure, with a full return to normalcy by the second day. We have now including this information and the citation in the Discussion section. Finally, we have not noticed any major changes in maternal behavior in the GIH mothers. For example, during the ultrasonic vocalization studies, we noticed that when the pups are returned to the cage with their mothers, the GIH mothers huddle all of the pups just like the GNX mothers do. One possible complicating factor would be if the GIH mothers do not nurse their pups properly, which could give rise to deficits in later life. To this end, we measured male and female GNX and GIH pups during the period they are under maternal care (i.e., up to 3 weeks of age). We found no differences in GIH pup weight or in pup weight gain during this period relative to GNX pups. More specifically, GIH male pups were within 4% of GNX male pups during this period, and GIH female pups within 2% of GNX female pups during this period. These findings and the associated statistics are now included in the first paragraph of the Results section.

Along the lines of mechanism, in response to comments from another reviewer (Reviewer 3), in this resubmission we identified a biochemical change occurring in the cortex of GIH male offspring, namely excessive levels of mTOR signaling. We then show that a pharmacological approach aimed at alleviating this hyper-mTOR activity is able to rescue the behavioral phenotypes of GIH male offspring. These new data comprise Main Figures 8 and 9.

Reviewer #2: This seems a revised manuscript, but I was not involved in previous review nor able to download the authors' responses. As such, I can only judge this manuscript as new. The results are solid and the discussion plus conclusion is appropriate.

We appreciate the enthusiasm for our first submission.

Reviewer #3: authors showed an interesting condition in which treatment for low oxygen of pregnant mouse will exhibit lasting effects in social behaviors in offsprings. they analyzed the social behaviors and potential cellular mechanisms involved, such as spine morphology and synaptic physiology. however, I do feel that although the data authors presented are carefully analyzed, the whole work seems too descriptive but lack of causal mechanisms. is there any molecular or cellular mechanism in any parts of brain would be account for this abnormalities? for example, is there any molecular or cellular manipulations would be able to rescue this effects caused by maternal condition? without causal connections, it is hard for this study to make important impact to the field.

We agree that a significant limitation of our first submission was the lack of a molecular/cellular mechanism that can explain the behavioral abnormalities we identified in the GIH male offspring. Further, we agree that the identification of a mechanism would strengthen our manuscript. To this end, during the resubmission period, we investigated potential biochemical processes that could contribute to the behavioral phenotypes of GIH male offspring. We initiated this process by examining changes in signal

transduction pathways in medial prefrontal cortex (mPFC) homogenates micro-dissected from 3 week old (juvenile) GNX and GIH offspring. We chose this time period as it is prior to the onset of major neuronal morphological changes in the GIH offspring, and thus any identified biochemical changes are less likely to be a mere consequence of these neuronal alterations than if we examined tissue from older rats. We focused our attention on the mTOR signaling pathway as this pathway has been repeatedly implicated in autism spectrum disorder. More specifically, mTOR pathway hyperactivity has been identified in the cerebral cortex in cases of idiopathic autism, and excessive engagement of the mTOR pathway is a consequence of several monogenic disorders that are associated with a greatly increased incidence of autism (e.g., tuberous sclerosis). Further, dampening the mTOR pathway has been proposed as a potential therapeutic approach in autism.

Our biochemical findings indicate that feedforward mTOR activity is excessive in the medial prefrontal cortex of juvenile GIH male offspring as compared to GNX offspring. As mTOR has many downstream targets that have a broad range of functions, we sought to investigate which, if any, mTOR target proteins are affected in GIH male offspring. Our findings indicate that the pro-autophagy kinase, ULK1, is the mTOR target most strongly affected in GIH male offspring. mTOR reduces neuronal autophagy by phosphorylating ULK1 at the serine 757 residue, thereby reducing the ULK1's activity and consequent ability to initiate autophagy. In GIH male offspring, we found increased levels of ULK1 phosphorylation at this residue, indicative of increased mTOR-mediated phospho-inhibition of ULK1. The phospho-activity of other mTOR targets that are not implicated in regulating autophagy, but rather have defined roles in controlling protein translation (e.g., 4E-BP1 and p70S6K), were not affected in GIH male offspring. Overall, these findings pinpoint a specific aspect of mTOR signaling as a potential contributor to the aberrant phenotypes in GIH male offspring. <u>These new biochemical data are shown in Main Figure 8.</u>

As our biochemical data implicate excessive mTOR signaling in GIH male offspring, we wanted to determine if dampening mTOR activity can alleviate the behavioral phenotypes of these rats. To this end, we implanted slow-diffusion pellets containing rapamycin (a well-characterized and specific inhibitor of mTOR) into the mid-scapular region of 3 week old GNX and GIH male rats; a vehicle pellet was used as a control. Rapamycin crosses the blood-brain barrier. After implantation, the pellets were formulated to last for 3 weeks (until the rats were 6 weeks of age), at which time rats were assessed for cognitive and social behaviors. First, we found that vehicle-treated GIH male offspring were impaired in all assessed cognitive and social tasks relative to vehicle-treated GNX male offspring, consistent with the findings from our fist submission that identified behavioral impairments in GIH male offspring. Importantly, we found that in GIH male offspring, rapamycin rescued deficits in Y-maze spontaneous alternation, novel object recognition, social approach, and preference for social novelty, relative to vehicle-treated GIH offspring. On the other hand, rapamycin impaired cognitive and social behavioral performance in GNX male rats. These findings indicate that rapamycin has opposite effects on behavior in GNX vs. GIH male offspring, as it is therapeutic for GIH male offspring, but is adverse for GNX male offspring. The likely reason for this difference is that GIH male offspring have excessive mTOR activity, and thus reducing this hyperactivity is likely beneficial; however, in male GNX offspring rats with normal pre-existing levels of mTOR activity, reducing this activity below baseline levels is detrimental. <u>These new data are shown in Main Figure 9.</u>

Taken together, these new data not only identify a critical biochemical change in the male GIH offspring mPFC, but also demonstrate the relevance of this biochemical alteration in contributing to the core behavioral aberrations.
